# The MRI-based 3D morphologic changes of knee meniscus under knee weight-bearing and early flexion conditions

**DOI:** 10.1038/s41598-021-01531-9

**Published:** 2021-11-11

**Authors:** Tong Liu, Xianyue Shen, Qingming Ji, Jianlin Xiao, Jianlin Zuo, Zhongli Gao

**Affiliations:** 1 Department of Orthopedics, China-Japan Union Hospital of Jilin University, Changchun, Jilin Province People’s Republic of China; 2 Department of Orthopedics, The Second Hospital of Jilin University, Changchun, Jilin Province People’s Republic of China; 3 Department of Intensive Care Unit, The First Hospital of Jilin University, Changchun, Jilin Province People’s Republic of China

**Keywords:** Anatomy, Medical research

## Abstract

There are few studies investigate morphologic changes of knee meniscus in vivo mechanical loading and three-dimensions (3D) deformation and displacement of the whole meniscus between in vivo mechanical loading and unloading conditions are still unclear. To investigate the displacements and 3D morphological changes of the menisci under knee weight-bearing and early flexion conditions in healthy adults using a Magnetic Resonance Imaging (MRI)-compatible loading device (a 3.0 T MR imaging system) combined with a newly developed 3D comparison technique. Fifteen healthy volunteers were recruited in this cross-sectional observational study. Each subject underwent MRIs of their dominant right knee in eight different scanning conditions using a 3.0-T MRI scanner with a custom-made MRI-compatible loading device. The knee meniscus images were 3D reconstructed, and dimensional comparisons were made for each meniscal model with baseline (0°-unloaded model). The morphologic changes of the meniscal-anterior horn (AH), body (BD), and posterior horn (PH) regions were expressed as mean positive and negative deviations. The displacements were further investigated, and the meniscal extrusions of different subregions were measured. The morphologic changing patterns of human meniscus under loading and flexions were presented using 3D chromatic maps. The bilateral menisci were generally shifting laterally and posteriorly in most flexion angles and were changing medially and anteriorly under fully extended knee loading conditions. The mean deviations were more significant with loading at 0° of knee flexion, while the PH region in the lateral side changed further posteriorly with loading in 30° flexion. Most of the differences were not significant in other flexion angles between loading conditions. The extrusion of meniscus’s medial body was greater in full extension compared to any other flexing angles. Mechanical loading can significantly deform the menisci in knee extension; however, this effect is limited during knee flexion. Current study can be used as a reference for the evaluations of the integrity in meniscal functions.

## Introduction

The meniscus is a crucial mechanical component of the knee responsible for loading transmission and shock absorption. The human menisci transmit 30–55% of the load in a standing position and 90% during knee flexion^1^. The knee meniscus is also known as a secondary stabilizer, which limits the excessive displacement in multiple knee joint directions with the combination of ligaments.

Magnetic Resonance Imaging (MRI) is one of the most effective tools for analyzing changes in musculoskeletal structures and related pathologies. Yet, during the MRI scan, patients are placed in a supine position, which may remarkably change the morphology of load transmission structures, such as meniscus and joint cartilage in the knee, compared to actual human’s physiological configuration^2,3^. To overcome this problem, mechanical loading devices and the corresponding methodologies were developed to investigate the human knee joint's anatomical status under actual or mimicking weight-bearing conditions. Jerban et al.^2^ reviewed MRI studies of the knee under loading from 1998 to 2020 revealing that MRI-compatible loading devices have been used to scan patients in a supine position providing enough magnetic field strength and maintaining relatively stable loading states during scanning. Using these methods, joint cartilage was investigated by multiple MRI measures; yet, only a few studies used this approach to investigate the meniscus. For example, Stehling et al.^4^ and Patel et al.^3^ compared the meniscal extrusion between axial loading and unloading conditions in healthy volunteers and knee osteoarthritis (OA) patients. They found significant increases in medial meniscal extrusion under loading conditions, especially in degenerated knees. Still, three-dimensions (3D) deformation and displacement of the whole meniscus between in vivo mechanical loading and unloading conditions are not clear. Also, the meniscal load-carrying status was proven to be most pronounced in the early stance phase of gait during daily human activities^5^. Since most of the studies were performed only in the fully extended knee, more research on early knee flexion^6^ (0°–30°) positions is needed as it could further our understanding of the physiological functional morphology of meniscus. Such loading-related anatomical information may also provide references for the evaluations of the integrity in meniscal functions.

In the current study, we investigated the displacements and 3D morphological changes of the menisci in healthy adults under knee weight-bearing and early flexion conditions using an MRI-compatible loading device, a 3.0 T MR imaging system, combined with a newly developed 3D comparison technique.

## Materials and methods

### Subjects

The current study was approved by the Institutional Review Board Ethics Committee of China-Japan Union Hospital of Jilin University (IRB No. 2016-nsfc028). All subjects signed informed consent before participation and all experiments were performed in accordance with relevant guidelines and regulations. The inclusion criteria of the volunteers were: no knee joint pain or instability, skeletally mature, and no history of moderate to the severe knee joint injury. The exclusion criteria were: Body Mass Index (BMI) ≥ 30 kg/m^2^, congenital meniscus dysplasia, meniscus-related lesions, diffuse cartilage damage, and preexisting cruciate ligament injuries. All enrolled volunteers underwent MRI of the dominant knee using a 3.0-T MRI scanner.

### MRI scanning

Each subject was required to take MRIs in eight different loading conditions using a 3.0-T MRI scanner (Siemens Healthineers, Erlangen, Germany) with a body 18 channel soft coil (Siemens Healthcare GmbH, Erlangen, Germany) to cover up to 30° of knee flexion. All scans were performed using T2-weighted double echo steady-state (DESS) water excitation (WE) sequences with the following parameters: echo time: 5.0 ms, repetition time: 14.8 ms, field of view: 14 cm, matrix: 256 × 240, slice thickness: 0.6 mm, number of excitations: 1, acquisition time: 4.0 min.

**Figure 1:**
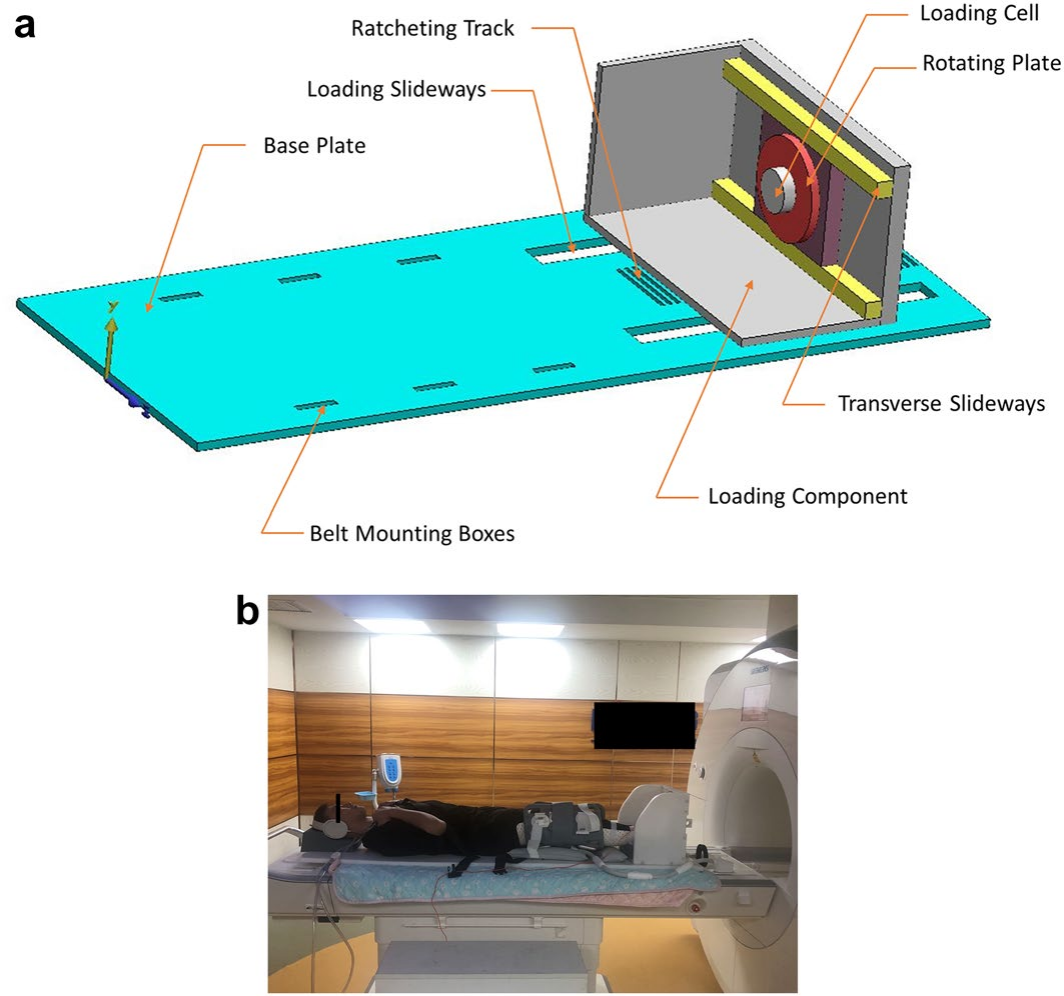
(**a**) The custom-made MRI compatible loading device was manufactured from Delrin (a nonmagnetic polymer), and it was consisted of a base plate and a loading component. The shoulder belts and waist straps were fixed in the mounting boxes on the base plate to hold the upper body of the tested subject. The loading component was connected to the base plate and the axial translation was permitted through the loading slideways using ratcheting system. A 6-axis load cell was placed on the loading component with a rotating plate and transverse slide tracks which helps for positioning of the tested extremities in different subjects. (**b**) A subject was placed on the loading device with a supine position. The foot of the subject’s testing lower extremity was stepped on the loading component, which can be used to generate axial mechanical loading with a ratcheting system. The loading cell mounted on this component was connected to a digital monitor, which can be spectated by the investigators and the subject to maintain a 50% BW condition during scanning. The subject’s upper body's position was constrained by shoulder belts and waist straps to prevent moving during loading. Specifically made polymer splints maintained the flexion angles of the testing knee.

Subjects were placed in a wheelchair 45 min before their first MRI scan to avoid the knees' weight-bearing. Afterward, the volunteers were positioned in a custom-made MRI compatible lower extremity loading device in a supine position with the tested dominant (all right) knee initially fully extended (Fig. 1a,b) (We confirmed that informed consent to publish was obtained from the participant depicted in Fig. 1b). Four polymer splints were specifically made for each subject to maintain their tested knee flexed in required angles (0°, 10°, 20°, and 30°). The flexion angles of the knee (the angle enclosed by the midlines of tibial and femoral shaft on sagittal plane) were also confirmed on positioning image before scanning. In addition, 50% subject body weight (BW) of axial force was applied on the tested leg by the loading device using a ratcheting system when a loading condition was acquired. There were totally eight MRI scans for each subject, including unloaded 0° flexion (Baseline), loaded 0° flexion, unloaded 10° flexion, loaded 10° flexion, unloaded 20° flexion, loaded 20° flexion, unloaded 30° flexion, and loaded 30° flexion. The volunteers were asked to have a 30 min rest time between each scan to decrease the impact of loading and flexion in the knee.

### Image analyses

The scanning data were deposited as Digital Imaging and Communications in Medicine (DICOM) format and were imported to Mimics 17.0 software (Materialise, Belgium) for 3D reconstruction of the menisci. The images of menisci were segmented using thresholding function with manual editing performed by an experienced surgeon. The 3D meniscal models were then generated and optimized by limited smoothing. The proximal 10 cm of the tibial plateau in each scan was 3D reconstructed in the same manner.

The 3D models obtained from every eight scans of each subject were imported into Geomagic Qualify 13.0 (Geomagic, Research Triangle Park, NC, USA). According to our previously established methodology^7,8^, the 3D comparisons were conducted in the current environment. The models were best fit aligned according to the tibial plateaus using the baseline (0°-unloaded) images as the reference for every subject. When the alignments were satisfied, dimensional comparisons were made between the baseline and the treated (with loading and/or knee flexion conditions) meniscal models for analyzing their morphological differences. The mean positive and negative differences, also called the positive/negative deviations, were the values in describing the changing direction and distance of the tested model. When the test surface is moving away from the reference, the difference is presented as a positive value. If the test surface is moving close to or even into the reference model, the difference value would be negative. In the current study, the medial and lateral menisci were further divided into the anterior horn (AH), body (BD), and posterior horn (PH) sub-regions for detailed explaining the changing directions of each section of the model.

**Figure 2:**
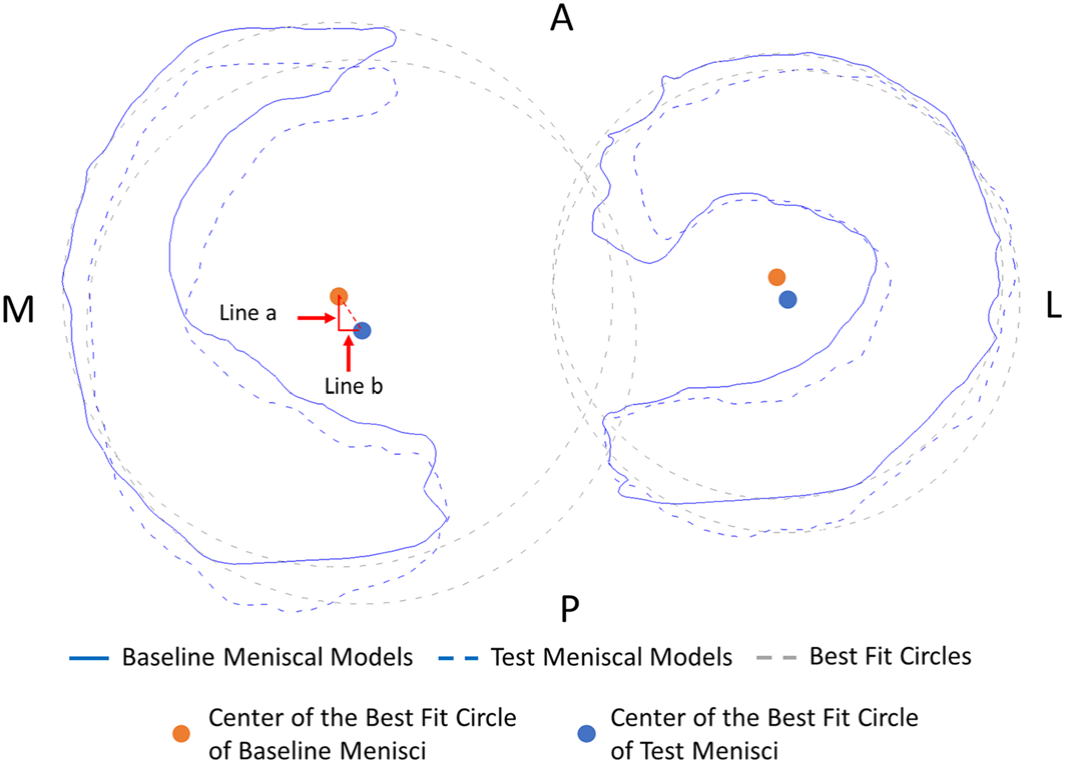
The example of anterior–posterior (Line a) and medial–lateral (Line b) displacement measurements of tested menisci.

The parameters above were used to describe the general morphological changes of each test meniscus. However, it was not clear if those changes were made either from displacements or from deformations. Therefore, the displacements of the test menisci were further measured based on our previously published method^9^. The aligned meniscal models were imported into 3-matic 7.0 (Materialise, Belgium) environments, and their corresponding 2D projections were obtained according to the tibial plateau plane. Then, best-fit circles were used to outline each of the projected menisci, and the circle centers were applied to indicate the models' 2D locations. On the 2D projected plane, the anteroposterior (AP) and medial–lateral (ML) directions were defined based on the intercondylar eminence line and its widest perpendicular line on the articular surface of the tibial plateau. The AP distance (Line a) and ML distance (Line b) between baseline and test centers of the meniscal best-fit circle were independently measured (Fig. 2), and anterior and lateral directions were set as positive.

Finally, the extrusion of meniscus out of the tibial plateau under different loading and knee flexion conditions was measured using a similar approach previously described in the literature^10^. In the current study, we used the radial midlines of each of the AH, BD, and PH regions. The protruding distances out of the tibial plateau's outermost edge on the midlines were measured as the meniscal extrusion distances.

### Statistical analyses

Statistical analyses were conducted in PASW Statistics 18.0 (IBM Inc., Armonk, NY, USA) software. The continuous data were presented as mean ± standard deviations. In the comparisons of positive/negative deviations, different knee flexion angles were analyzed by either ANOVA (parametric data) or Friedman test (nonparametric data). The comparisons were made for every combination of the lateral/medial meniscus, the AH/BD/PH subregion, the positive/negative deviation, and the unloaded/loaded condition, respectively. The meniscus' displacements were assessed using ANOVA or Friedman test for different flexion angles in each of the meniscus groups and the loading conditions. The meniscus' extrusions were compared among 0 to 30° of knee flexions with ANOVA or Friedman test for any subregions of lateral and medial menisci, respectively. Finally, between loading conditions, the deviations, the displacements, and the extrusions of meniscus were further compared with paired Student t-test or Wilcoxon signed-rank test if the data were nonparametric; AP and ML displacements of the same conditions were also compared. For all the analyses above, p < 0.05 was set to be statistically significant. The intraclass correlation coefficient (ICC) was evaluated by a one-way random effects model to quantify interobserver and intraobserver reliability of the extrusion and displacement measurements to assess the reproducibility. Power analyses were performed for meniscal extrusion and displacement between loading groups based on our pilot tests using G*Power 3.1.9.7 (Medistat, Kiel, Germany) software. The mean and standard deviation were calculated. With an effect size of 0.80, an α of 0.05, and 15 subjects in each group, the power to detect the difference of meniscal extrusion is 0.82; with an effect size of 0.74, an α of 0.05, and 15 subjects in each group, the power to detect the difference of meniscal displacement is 0.76.

## Results

17 volunteers were recruited according to the inclusion criteria, and two of them who met the exclusion criteria (one with preexisting medial meniscus grade 2 horizontal tear; one with preexisting tear in anterior cruciate ligament) were excluded from this study. Finally, 15 healthy volunteers (all male, mean age 25.4 years [range, 23–28 years]) were enrolled in this study (Table 1).

**Table 1:** Demographic data of enrolled subjects.

Subjects	Gender	Height (m)	Weight (kg)	BMI	Age	Dominant side
Subject01	Male	1.80	84.00	25.93	26	Right
Subject02	Male	1.75	60.00	19.60	24	Right
Subject03	Male	1.81	69.00	21.06	25	Right
Subject04	Male	1.78	63.00	19.88	26	Right
Subject05	Male	1.68	58.00	20.55	25	Right
Subject06	Male	1.92	90.00	24.41	25	Right
Subject07	Male	1.77	65.00	20.75	24	Right
Subject08	Male	1.73	70.00	23.39	23	Right
Subject09	Male	1.80	83.00	25.62	24	Right
Subject10	Male	1.80	74.00	22.84	28	Right
Subject11	Male	1.80	80.00	24.69	27	Right
Subject12	Male	1.74	62.00	20.48	25	Right
Subject13	Male	1.82	75.00	22.64	26	Right
Subject14	Male	1.78	74.00	23.36	27	Right
Subject15	Male	1.65	62.00	22.77	26	Right

*BMI* body mass index.

**Figure 3:**
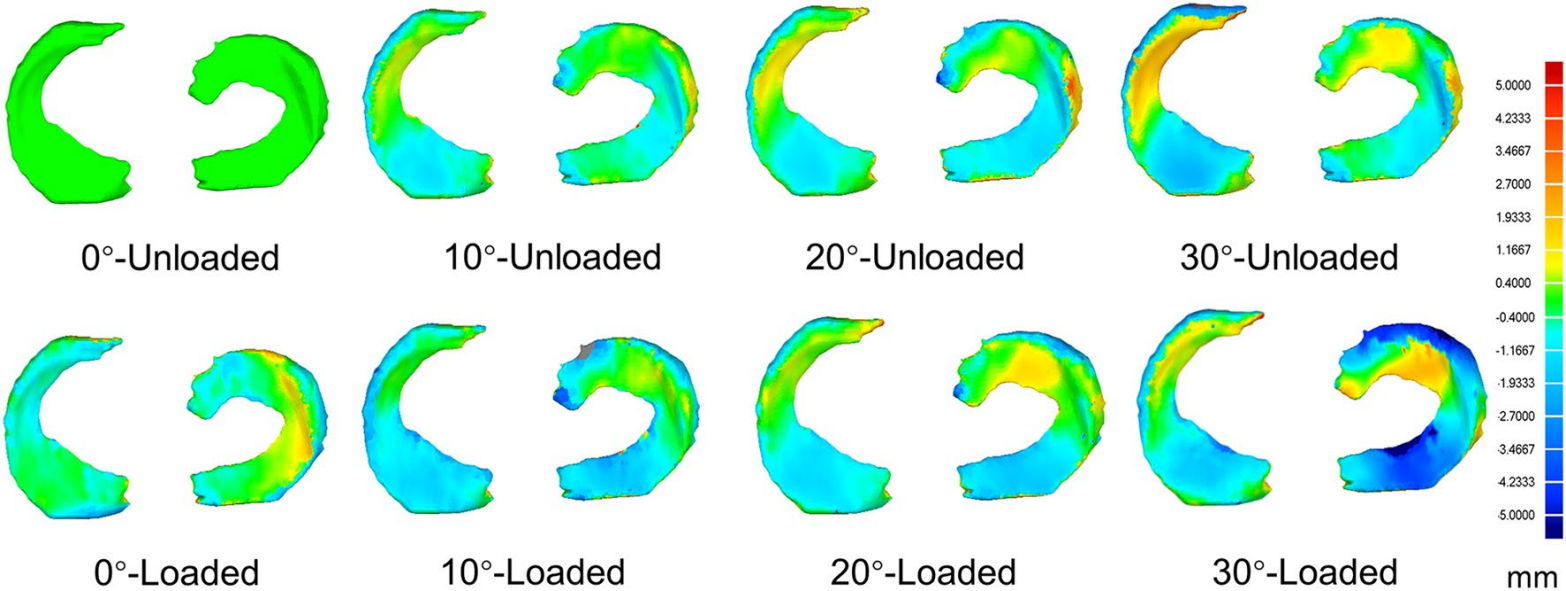
The general change color maps of a subject in Geomagic Qualify software 13.0 (Geomagic, Research Triangle Park, NC, USA). The scale bar in the right was applied to all the color maps with the unit in millimeters.

The examples of the dimensional comparison chromatic graphs in Geomagic Qualify 13.0 (Geomagic, Research Triangle Park, NC, USA) for the general changes of one single subject are shown in Fig. 3.

Based on the unloaded-0° flexion baseline model, in the medial meniscus of loaded-full extension condition, the AH and PH were shifting anteriorly, the BD was shifting medially, and the top surfaces of AH and BD were compressed down. In this model’s lateral side, the AH and PH were shifting forward and backward, respectively; the BD was shifting medially, and the AH and PH were compressed down as well. In 10°, 20°, 30° knee flexion menisci with and without loading, the general changes were similar: the AH was moving posteriorly, the BD was moving laterally, and the PH was compressed and shifted backward in the medial meniscus; for the lateral side, the AH and PH were shifting posteriorly, the BD was moving laterally, and the BD and PH were both compressed. The other 14 subjects shared the same patterns in both general and shape changes. According to the color maps, the changes in 30° of flexion were greater than the models at 10° and 20°, and the loaded meniscus was basically associated with greater changes than unloaded.

The quantified comparisons are summarized in Appendix Table A1a,b. Differences among flexion angles in unloaded conditions and between loadings in extension were significant (p < 0.05). The PH was changing further posteriorly (p < 0.05) under loading in lateral side when the knee flexed in 30°. The displacements of menisci under various loading and flexion conditions are shown (Appendix Fig. A1a,b). When unloaded, the menisci of both sides were shifting laterally during early knee flexions, and both of the menisci were moving outwards when the knee was loaded and fully extended (lateral: 0.68 ± 2.53 mm; medial: − 0.94 ± 1.40 mm). In most of the conditions, the menisci were moving backward for the A-P direction due to knee flexion and loading. Comparison of the results of meniscal displacements are shown in Appendix Table A2. Significant increases in lateral shifting (30°-unloaded lateral side: 2.01 ± 1.75 mm) and posterior shifting (30°-unloaded lateral side: 2.81 ± 2.41 mm; 30°-loaded lateral side: 3.54 ± 2.26 mm; 30°-unloaded medial side: 1.18 ± 0.89 mm) were revealed among all investigated flexion angles. The AP displacements were significantly greater than those of the ML direction in both lateral (1.84 ± 2.18 mm vs. 1.06 ± 1.90 mm, p < 0.01) and medial menisci (0.53 ± 1.04 mm vs. 0.03 ± 1.22 mm, p < 0.01). In the meniscal extrusion measurements, the medial AH and BD extruded mostly in the full extension position than any other flexion angles, but only the extrusion of the loaded body reached statistical significance (2.93 ± 1.36 mm, p = 0.044). The PH was not extruding in most conditions (Table 2). No extrusion was noticed in the lateral meniscus based on our methodology. Intraclass correlation coefficient results of the intraobserver and interobserver reliabilities were both > 0.75, which were in the excellent range (Table 3).

**Table 2:** Extrusion of medial meniscus.

	0°	10°	20°	30°	*p*
**Anterior horn (mm)**					
Unloaded	3.43 ± 1.26	3.17 ± 1.38	2.88 ± 1.42	2.55 ± 1.43	0.34
Loaded	3.57 ± 1.38	2.93 ± 1.24	2.35 ± 1.55	2.33 ± 0.86	0.03*
*p*	0.79	0.63	0.33	0.62	
**Body (mm)**					
Unloaded	1.90 ± 0.68	2.01 ± 1.02	1.86 ± 0.90	1.97 ± 1.08	0.97
Loaded	2.93 ± 1.36	1.90 ± 0.63	2.17 ± 0.67	1.91 ± 1.17	0.02*
*p*	0.01*	0.55	0.29	0.75	

Data are presented as mean ± standard deviation. *p < 0.05.

**Table 3:** Intraobserver and Interobserver reliability for meniscal deformation measurements.

Measurements	Intraobserver intraclass correlation coefficient	Interobserver intraclass correlation coefficient
Extrusion AH	0.973 (0.962–0.981)	0.949 (0.928–0.964)
Extrusion BD	0.962 (0.946–0.973)	0.960 (0.943–0.972)
Lateral displacement	0.774 (0.691–0.837)	0.767 (0.682–0.832)
Medial displacement	0.883 (0.827–0.921)	0.862 (0.797–0.907)

Data are presented as intraclass correlation coefficients, with 95% confidence interval in parentheses.

## Discussion

The term “Meniscus” is originally from a Greek word, which means crescent shaped. In physics, “Meniscus” refers to the crescent shaped curve of the liquid surface close to the wall of the container, and its interesting microstructures had been further investigated by Li et al.^11^. The crescent shaped cartilage in human knee joint is also defined as “Meniscus”, and critical biomechanical functions are carried by this important structure. The normal human knee meniscus's morphological changes in 0 to 30 degrees of knee flexions with and without mechanical loading were examined in the current study. The general change expressed in the study indicated the combination of displacement and the shape change of meniscus (Fig. 3). The lateral and medial menisci were generally shifting laterally and posteriorly during knee flexion in both loaded and unloaded conditions, except the medial menisci were shifting slightly to the medial side (Appendix Table A2) when the loading was applied to fully extended knee, which was consistent with results reported by Tienen et al.^12^. This finding can be attributed to the increased joint-capsule laxity^13^ and tibial external rotation during knee early flexion, which makes the meniscus moving posteriorly. Using different experimental approaches, Vedi et al.^6^ and Tienen et al.^12^ proved that the AP displacements are greater than ML direction in the menisci with knee flexion and during the loaded or unloaded condition. In the present study, we assessed the morphologic changes in lateral and medial menisci, and we found similar results on both sides. This result could be explained as the movements of femoral condyle on tibial plateau are limited in ML than in AP direction. Still, in this study, we only investigated the meniscal changes in 0 to 30 degrees of knee flexion due to the current methodology's limitation; thus, meniscal changes in deep knee flexion remain unclear. In this study's displacing measurements, we used the center of the meniscal best-fit circle to indicate the 2D position of the meniscus as a whole. By measuring the translations among the centers under different conditions, the meniscal displacements were obtained (Fig. 2). Significant posterior shifting was shown in unloaded menisci during 30°knee flexion compared with baseline, and significant lateral shifting was observed in unloaded lateral menisci at 30° flexion. Moreover, only the loaded lateral meniscus presented greater posterior shifting; for other conditions, the AP or ML direction difference was not significant (Appendix Table A2). Based on these results, it should be noted that the menisci moved remarkably during knee flexion, while static loading may result in even greater shifting with limited effects.

Several studies analyzed the morphological changes in menisci's anterior horn, body, and posterior horn. Vedi et al.^6^ found that anterior and posterior horns of meniscus move posteriorly during knee flexion with or without loading, while the body sections move medially or laterally in medial or lateral meniscus, respectively. In addition, they found that the moving distances were greater in a loaded condition; yet, only the anterior horn of the lateral side reached significant difference. In the current study, we used the mean deviations calculated from 3D comparing models instead of the 2D measurements on a single MR slice to describe the regional morphologic change of meniscus. This approach can help understand each section's general changing patterns rather than the analyzing of limited pixels. For each region, the positive and negative mean deviations were calculated to determine the 3D performances of the models since the meniscus is not rigid and the difference between positive/negative morphologic changes should exist. Significant differences were associated with knee extension between loaded and unloaded scanning in every meniscal region, suggesting the strong contribution of mechanical loading in the meniscus' morphologic changes. Yet, such significance was limited during knee flexions between loading conditions (Appendix Table A1), and only the posterior horn in 30° flexion exhibited remarkable posterior change. For the unloaded menisci in the lateral side, the changing values were similar or even smaller at 20°of flexion compared to 10°, and the changes further increased in 30° of knee flexion. Comparatively, approximate linear increases of changes were shown with loading, but only the lateral posterior horn in 20° of flexion was significantly different. When knee flexed to 30 degrees, bilateral menisci's changes increased under loading conditions, which was consistent with previously published data^6,12,14^. The reason of such difference was unknown, but one potential explanation could be excessive posterior sliding of femoral condyle generated with mechanical loading during knee flexion, and this may further lead to greater translation and deformation on the posterior horn of meniscus.

The meniscus' extrusion is considered as a reliable indicator of knee osteoarthritis (OA), meniscal degeneration, and root tears. Achtnich et al.^15^, Karpinski et al.^16^, and Shimozaki et al.^17^ used MRI and ultrasound techniques to investigate the meniscal extrusions with and without loading in healthy volunteers and patients with meniscus injuries, identifying significantly greater extrusions in medial meniscus during loading conditions. By applying a similar testing approach as used in the present study, Stehling et al.^4^ and Patel et al.^3^ compared the medial meniscal extrusion between healthy adults and OA patients and found that higher extrusion was associated with mechanical loading, especially in subjects with knee OA. This study found that the medial meniscal extrusion was significantly greater in the body section only with the knee fully extended during loading. The extrusions of the anterior meniscal horn, posterior horn, and the body in flexed knee were also measured; yet, no statistical difference was found between loading conditions. In Stehling’s study^4^ and Achtnich’s study^15^, authors examined the 20°flexion instead of full extension for bearing of the mechanical loading. This method may help subjects to maintain the strength of loading during scanning. Yet, according to the literatures^6,12,14^ as well as our results the menisci is moving laterally during scanning, and the meniscus extrusion measured in such angle may lead to underestimated values compared with the testing of the knee in full extension position. It was also confirmed in our study that the extrusion of medial meniscus in 20° knee flexion was significantly lower than that in fully extended knee (2.17 ± 0.67 vs 2.93 ± 1.36 mm). No extrusion was detected in the lateral meniscus in any circumstance, which is consistent with Wenger et al.^10^.

Different in vitro and in vivo approaches have been developed to investigate the human meniscus’ morphologic changes. Using isolated meniscal specimens, Freutel et al.^18^ and Fowlie et al.^19^ investigated the deformation and displacement of the meniscus with MRI under in vitro simulated knee motion. Nishimuta and his team^20,21^ summarized the strains under different laboratory loading conditions with the research materials. Moreover, Kessler et al.^22^ reported that the changes of strains were associated with different regions in the meniscus and suggested that higher strain may be a source of meniscal degeneration. Hauch and colleagues^23^ investigated meniscal attachments' mechanical performances under loading and found the differences in strain using isolated human menisci. Said et al.^24^ and Schad et al.^25^ applied customized mechanical devices and MRI scanning to examine the cadaveric human knee joint's in-situ alignments under Varus-valgus and compressive loadings.

With reference to in vivo work, Jerban et al.^2^ comprehensively reviewed the mechanical loading in vivo studies of knee joint based on MRI. The authors pointed out that compared with the low magnetic field strengths provided by open-bore interventional MRIs, the combination of a supine loading device with ordinary MRI is more often applied to explore knee articular cartilage performances meniscus under loading. In the current study, we used a custom-made MRI-compatible knee loading device with a 3.0 T MRI scanner to obtain preferable image resolutions, maintaining demanded loading strengths during scanning. The loading devices applied in the previous studies were also compared by Jerban et al. The concept of ratcheting mechanism suggested by Wang et al. and Maher et al.^26,27^ was applied in the current study to generate mechanical loading during MR scanning, and polymer splints were newly introduced to stabilize the knees during scanning in flexed positions. The manner above was applied in only a few studies in meniscus^3,4^. To our knowledge, this is the first study to investigate the 3D morphologic change of human meniscus under loading and knee flexion conditions using in vivo MRI-compatible loading device.

This study has a few limitations. First, the sample size in the current study was relatively small. However, since eight MRI scans for each subject were required according to the study protocol, and the total exam duration of a single volunteer was around 6 h, including rest time, it is impractical to achieve a large enough sample size using of clinically occupied MR scanner. Also, the statistical powers in the comparisons of meniscal extrusion and displacement were acceptable. Second, static loading conducted in this work by applying the supine loading method is not the ultimate approach as dynamic loading in the mimicking of the human knee joint's daily activities. Yet, an eligible meniscal 3D model for dimensional comparing required high enough resolutions of scanning image to fulfill the elaborative reconstructions with details. The current method was the best utilizable option for us. Third, this study's polymer splints for stabilizing knee flexion angle during scanning may produce extra mechanical support and may subsequently affect the measuring results. The limitations of the lacks of scanning data during knee flexion have been long-standing^26,27^, and by using the combination of a soft body coil with clinical splints, the researchers were able to earn a stable scanning status with limited costs, therefore we believe such potential limitation could be accepted. Finally, the knee flexion angle greater than 40° could not be fitted in the current MRI scanner, and novel solutions are still needed to understand dimensional meniscal changes in mid to deep flexions of the knee.

## Conclusion

This study used 3D displacement and morphological change of normal human knee meniscus under loaded and 0–30° knee flexion conditions using in vivo mechanical loading and MRI scanning methods. During flexions, the morphologies of bilateral menisci changed laterally and posteriorly; on the contrary, significant medial and anterior meniscal changes were associated with the fully extended knee loading. Also, the extrusion in the body of medial meniscus was greater under loading with knee extended comparing with knee flexions. To sum up, this study revealed the general meniscal changing patterns of the healthy knee during loading and 0°–30° flexion conditions, thus provided references for the evaluations of meniscal integrity in biomechanical functions. Also, it would be a basic study for further investigation of the pathology of human meniscus. Improvements on the methodology in the management of knee flexion angles are needed to facilitate future research.

## Supplementary Information

Supplementary Information.
