# Supplementary material for: The MRI-based 3D morphologic changes of knee meniscus under knee weight-bearing and early flexion conditions
Source: Sci Rep. 2021 Nov 11;11:22122. doi: 10.1038/s41598-021-01531-9 (PMC8586250; doi:10.1038/s41598-021-01531-9)
Supplement: Supplementary file 1 — Supplementary Information. [file 41598_2021_1531_MOESM1_ESM.docx]

Appendix Figure A1a-b: The displacements of lateral (a) and medial (b) menisci were shown for both unloaded and loaded conditions in 0-30 degrees of knee flexion. The detailed statistical data of comparisons are presented in Appendix Table A2.


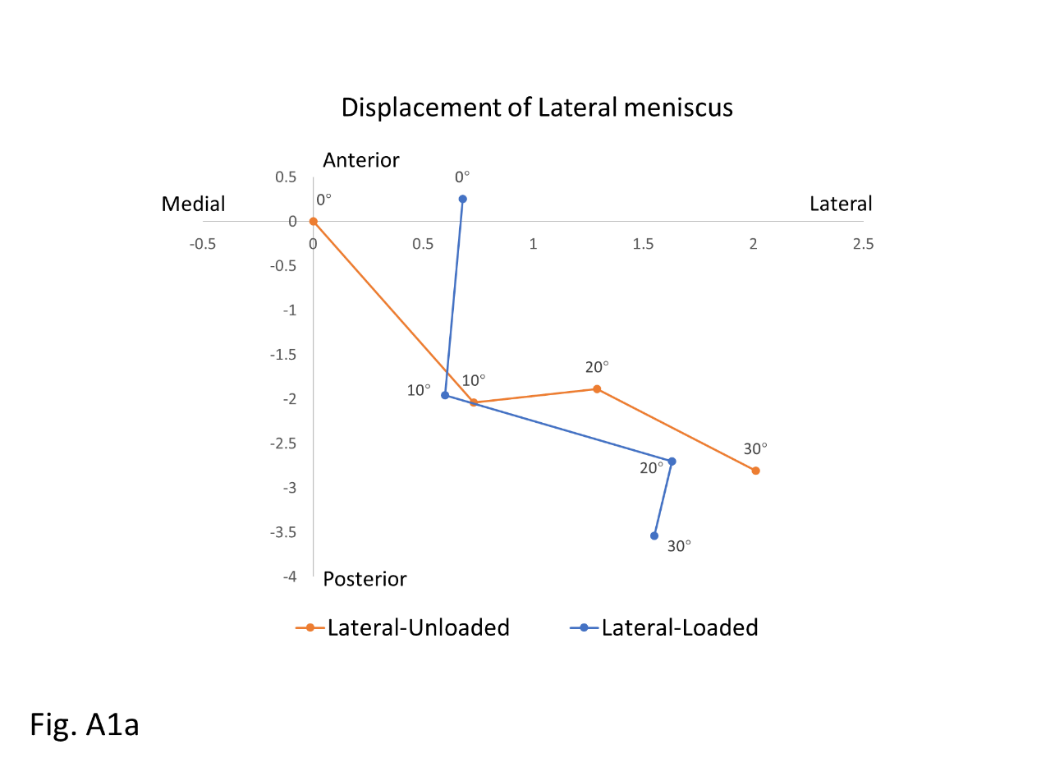

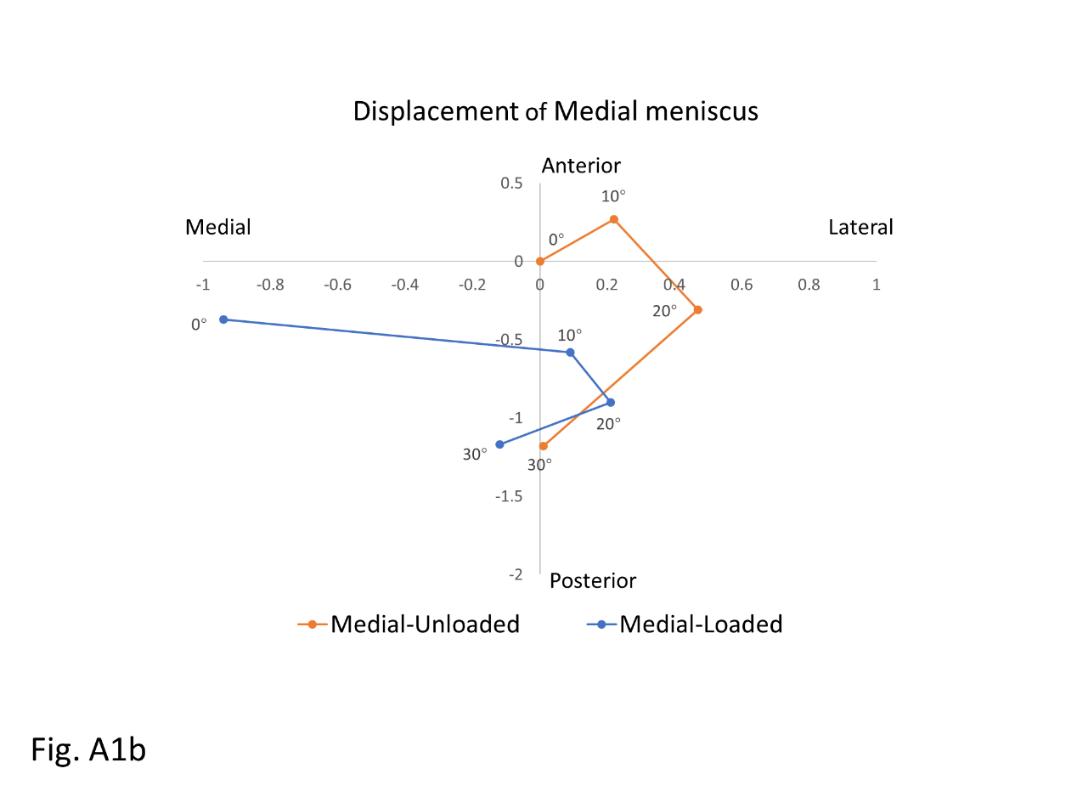


**Appendix Table A1a.** General Changes of Lateral Meniscus

|  |  | Anterior Horn(mm) | | | | |  | Body(mm) | | | | |  | Posterior Horn(mm) | | | | | |
| --- | --- | --- | --- | --- | --- | --- | --- | --- | --- | --- | --- | --- | --- | --- | --- | --- | --- | --- | --- |
|  |  | ***0°*** | ***10°*** | ***20°*** | ***30°*** | *p* |  | ***0°*** | ***10°*** | ***20°*** | ***30°*** | *p* |  | ***0°*** | ***10°*** | ***20°*** | ***30°*** | *p* |  |
| Mean Positive Difference | **Unloaded** | 0 | 2.21±2.08 | 1.56±1.06 | 3.19±2.62 | *<0.01** |  | 0 | 1.42±1.03 | 1.35±1.09 | 2.25±1.24 | *<0.01** |  | 0 | 1.35±0.74 | 1.91±1.64 | 1.93±1.69 | *<0.01** |  |
|  | **Loaded** | 1.04±0.58 | 2.18±1.90 | 2.57±1.67 | 3.38±2.53 | *<0.01** |  | 1.71±2.01 | 1.22±0.69 | 2.10±1.39 | 2.13±1.34 | *0.28* |  | 1.16±0.80 | 1.93±1.40 | 2.55±2.14 | 2.77±2.02 | *0.05** |  |
|  | *p* | *<0.01** | *0.95* | *0.01** | *0.70* |  |  | *<0.01** | *0.47* | *0.05** | *0.71* |  |  | *<0.01** | *0.13* | *0.03** | *0.04** |  |  |
| Mean Negative Difference | **Unloaded** | 0 | -2.37±1.39 | -1.95±1.31 | -3.27±2.54 | *<0.01** |  | 0 | -1.38±0.98 | -1.29±1.07 | -1.34±1.06 | *<0.01** |  | 0 | -1.53±1.68 | -2.16±1.65 | -2.30±2.09 | *<0.01** |  |
|  | **Loaded** | -0.98±0.83 | -2.21±2.33 | -2.27±2.35 | -3.43±2.72 | *0.03** |  | -1.51±1.84 | -1.25±1.07 | -1.32±1.03 | -1.57±1.10 | *0.90* |  | -1.04±0.89 | -2.25±1.16 | -3.08±2.12 | -3.99±2.47 | *<0.01** |  |
|  | *p* | *<0.01** | *0.75* | *0.53* | *0.61* |  |  | *<0.01** | *0.62* | *0.95* | *0.43* |  |  | *<0.01** | *0.10* | *<0.01** | *<0.01** |  |  |

Data are presented as mean±standard deviation. * p<0.05

**Appendix Table A1b.** General Changes of Medial Meniscus

|  |  | Anterior Horn(mm) | | | | |  | Body(mm) | | | | |  | Posterior Horn(mm) | | | | | |
| --- | --- | --- | --- | --- | --- | --- | --- | --- | --- | --- | --- | --- | --- | --- | --- | --- | --- | --- | --- |
|  |  | ***0°*** | ***10°*** | ***20°*** | ***30°*** | *p* |  | ***0°*** | ***10°*** | ***20°*** | ***30°*** | *p* |  | ***0°*** | ***10°*** | ***20°*** | ***30°*** | *p* |  |
| Mean Positive Difference | **Unloaded** | 0 | 1.38±0.68 | 1.91±1.25 | 2.71±1.31 | *<0.01** |  | 0 | 0.68±0.66 | 0.74±0.56 | 1.16±0.64 | *<0.01** |  | 0 | 0.74±0.55 | 1.22±0.97 | 1.14±0.57 | *<0.01** |  |
|  | **Loaded** | 0.93±0.44 | 1.18±0.66 | 1.95±1.31 | 2.41±1.64 | *<0.01** |  | 1.03±1.24 | 0.53±0.35 | 0.73±0.46 | 0.98±0.77 | *0.27* |  | 1.24±0.75 | 1.24±0.79 | 1.77±1.91 | 1.46±1.14 | *0.60* |  |
|  | *p* | *<0.01** | *0.27* | *0.93* | *0.41* |  |  | *<0.01** | *0.38* | *0.96* | *0.21* |  |  | *<0.01** | *0.08* | *0.24* | *0.35* |  |  |
| Mean Negative Difference | **Unloaded** | 0 | -0.67±0.39 | -1.01±0.81 | -1.30±1.13 | *<0.01** |  | 0 | -1.22±1.51 | -1.99±1.75 | -2.22±2.24 | *<0.01** |  | 0 | -1.16±1.34 | -0.85±0.51 | -1.51±1.31 | *<0.01** |  |
|  | **Loaded** | -0.91±1.36 | 0.59±0.37 | -1.00±1.49 | -1.26±0.99 | *0.46* |  | -1.78±1.88 | -1.64±1.50 | -2.21±2.02 | -2.34±2.31 | *0.72* |  | -1.14±0.62 | -1.08±0.72 | -2.11±2.01 | -2.13±20.2 | *0.09* |  |
|  | *p* | *0.02** | *0.54* | *0.99* | *0.81* |  |  | *<0.01** | *0.31* | *0.53* | *0.81* |  |  | *<0.01** | *0.87* | *0.02** | *0.03** |  |  |

Data are presented as mean±standard deviation. * p<0.05

**Appendix Table A2.** Displacement of Meniscus

|  |  | Lateral Meniscus(mm) | | | | |  | Medial Meniscus(mm) | | | | |
| --- | --- | --- | --- | --- | --- | --- | --- | --- | --- | --- | --- | --- |
|  |  | ***0°*** | ***10°*** | ***20°*** | ***30°*** | *p* |  | ***0°*** | ***10°*** | ***20°*** | ***30°*** | *p* |
| Anterior (+) - Posterior (-)  Shift | **Unloaded** | 0 | -2.04±1.92 | -1.89±1.58 | -2.81±2.41 | *<0.01** |  | 0 | 0.27±0.91 | -0.31±1.07 | -1.18±0.89 | *<0.01** |
|  | **Loaded** | 0.25±1.03 | -1.96±2.09 | -2.70±2.14 | -3.54±2.26 | *<0.01** |  | -0.37±0.90 | -0.58±1.02 | -0.90±1.36 | -1.17±0.81 | *0.18* |
|  | *p* | *0.37* | *0.86* | *0.03** | *0.05** |  |  | *0.13* | *0.01** | *0.21* | *0.97* |  |
| Lateral (+) -Medial (-)  Shift | **Unloaded** | 0 | 0.73±1.42 | 1.29±1.89 | 2.01±1.75 | *<0.01** |  | 0 | 0.22±1.27 | 0.47±1.28 | 0.01±1.56 | *0.67* |
|  | **Loaded** | 0.68±2.53 | 0.60±1.42 | 1.63±2.23 | 1.55±2.34 | *0.42* |  | -0.94±1.40 | 0.09±0.63 | 0.21±1.40 | -0.12±1.14 | *0.06* |
|  | *p* | *0.31* | *0.77* | *0.43* | *0.24* |  |  | *0.02** | *0.29* | *0.36* | *0.66* |  |

Data are presented as mean±standard deviation. * p<0.05
